# Prevalence of malaria and associated factors in Benna Tsemay district of pastoralist community, Southern Ethiopia

**DOI:** 10.1186/s40794-016-0033-x

**Published:** 2016-08-30

**Authors:** Gidie Woju Debo, Dejene Hailu Kassa

**Affiliations:** 1 UNICEF SNNPR Field Office, UNDP/UNV/UNICEF–Ethiopia, Hawassa, Ethiopia; 2 School of Public and Environment Health, College of Medicine and Health Sciences, Hawassa University, Hawassa, Ethiopia; 3 P.O.B. 12038, Addis Ababa, Ethiopia

**Keywords:** Malaria, Prevalence, Associated factors, Traditional practices, Pastoralist community

## Abstract

**Background:**

Though the burden of malaria is declining, challenges in control continue globally, regionally and nationally as the transmission of malaria is dynamic and determinants differ by place and time, and across populations. The current level of knowledge on malaria prevalence and associated factors in specific communities, such as pastoralist communities of Ethiopia, is lacking.

**Methods:**

A community based cross-sectional survey was conducted among pastoralist communities from December 2011 to January 2012. Background information and peripheral blood samples were collected from 461 randomly selected study participants. Multivariate regression analysis was done to explore the risk factors associated to malaria.

**Result:**

The prevalence of malaria among 461 examined study participants was 6.1 % (95 % CI = 4.2, 8.5). The infection rate with *Plasmodium falciparum* and *Plasmodium vivax* was 64.3 % (95 % CI = 45.5, 80.2) and 21.4 % (95 % CI = 19.8, 54.5), respectively, while mixed infection was 14.3 % (95 % CI = 4.7, 30.9). The infection rate was higher among lactating (22.2 %, 95 % CI =7.5, 45.3) and pregnant (17.6 %, 95 % CI = 4.7, 40.9) women compared with other community groups like infants (12 %). Mosquito net coverage of the study population was 90.1 % with an average of 1.8 per household. Traditional practices related to malaria prevention and treatment were not significantly associated with malaria infection (*p* > 0.05). Pregnancy (adjusted Odds Ratio [AOR]: 12.6, 95 % CI = 1.7, 94.7) and saving mosquito net for later use (AOR 9.6: 95 % CI = 2.2, 42.8) were independently associated with prevalent malaria infection.

**Conclusion:**

In spite of high coverage of mosquito nets, prevalence of malaria in this pastoralist community was high, affecting pregnant and lactating women at a higher rate. Pregnancy and saving mosquito nets for later use were identified as the associated risk factors. Health education on prevalence of malaria and knowledge on risk factors might be able to change the health behavior in this pastoralist community which consequently can decrease the malaria morbidity and mortality.

**Trial registration:**

ISRCTN ISRCTN73824458, Registered 28 September 2014.

## Background

Malaria has been a global challenge for many years [1, 2]. Evidence shows that about 3.3 billion people in the world were at risk of malaria in 2010. In the same year, the total deaths and cases attributed to malaria were 665,000 and 216 million, respectively. Among these, 91 % of the deaths and 81 % of cases occurred in African nations [1]. However, in 2010 country specific studies in some African countries revealed that the prevalence of malaria began to decline. For instance, in Uganda, the prevalence of malaria declined from 43 % (urban and rural) in 2004 to 23 % in rural and 3 % in urban areas in 2010 [2]. In Rwanda, malaria deaths fell by 67 % while cases fell by 55 % among children under five in 2007 [3].

Malaria in Ethiopia has remained a public health problem; even though, its burden has been declining following the mass distribution of Long Lasting Insecticide Treated Nets (LLITNs) and Arthemisine Combined Therapy (ACT) [4–8]. As the result of decentralization of interventions at the community level [9–15], a significant decline of malaria prevalence (4.1 % in 2006 to 0.4 % in 2007) was observed in a year interval [8]. There was a variation in prevalence from place to place and time to time across the nation, being very high among children and in all age groups where there were no sufficient interventions [3–7]. The average prevalence of malaria in the three highest populated regions (Oromiya, Southern Nation Nationalities and Peoples Region (SNNPR) & Amhara) was 4.1 %, the highest being in SNNPR (5.4 %) and the lowest in Oromia (0.9 %) in 2007 [8]. Prevalence of malaria was also identified with the variation of altitude, climate change, ecology of the environment, socio-economic status, parasite and vector control measures, and knowledge, attitudes and practices (KAP) among individuals towards interventions [4, 5, 8, 15–22].

Major challenges in controlling the spread of malaria include the vector’s resistance to Indoor Residual Spray (IRS) and the parasite to drug therapy [8, 21, 22]. Additional challenges consists of insufficient studies, inadequate surveillance, improper mosquito net (MN) use and the difference of malaria transmission & determinants dynamism from place to place in Ethiopia [8–11, 18–20]. Furthermore, few studies focus on factors of traditional practices related to malaria and no studies consider malaria control and risk in the remotest Ethiopian communities such as pastoralists who are vulnerable due to their nature of mobility [13–15]. Therefore, this study aims to assess the prevalence of malaria and associated factors constituting traditional practices and mosquito net use in a pastoralist district of southern Ethiopia.

## Methods

A community based cross-sectional study was conducted from December 2011 to January 2012 in Benna Tsemay district, Southern Ethiopia. The district is located 735 kilometers south west of Addis Ababa and the population is estimated to be 62,362 whose livelihoods are based primarily on rearing animals (pastorial). The altitude of the district is 1500 m above sea level with average annual temperatures ranging from 26 °C (during cold months) to 40 °C (during dry months). The rainfall distribution is bimodal with average annual rainfall of 800 mm. The district has a long dry season from December to the beginning of March, while June and July is a short dry season [23]. Traditional practices related to malaria treatment and prevention includes: ‘Boronge’, ‘Ara’, ‘Dugo’, witchcraft and ‘Rae/Rhanto’. ***‘Boronge’***- is a practice of giving the leaf juice of *‘Tseeako’* (locally named) herb for malaria (locally known as *Chinkilo)* patient every morning or night till the symptoms subside. ***‘Ara’***- is a practice of applying freshly slaughtered small intestine of a goat or sheep on the face of an acutely sick *chinkilo* patient and feeding the patient the meat***. ‘Dugo’*** – is a mechanism of blowing mosquitoes out from inside of a ‘*dugo’* house (traditionally built very small, conic in shape and covered by grass externally and internally laminated with a hide of tree or animal skin)***. Witchcraft*** – a consultation of witches to perform a magic healing for *chinkilo* patient***. Rhanto/Rhae*** – is a practice of piercing *chinkilo* patient’s frontal vein to get the dark blood out thinking that it could have caused the disease.

*A priori* sample size was estimated with the assumption of 50 % prevalence of malaria, 95 % confidence level and 5 % margin of error with a design effect of 1.2 for compensation of loss of efficiency [14]. Among the 28 kebeles (clusters) found in the district, 6 kebeles were selected using simple random sampling technique as a primary sampling unit. In each cluster (kebele), there were four villages (state teams), each with an average of 40 households (HH). Therefore, six clusters yield a total of 24 villages (6 clusters by 4 villages) and 960 estimated households (40 HHs by 24 villages). These villages were considered as the secondary sampling units; of which six villages were selected randomly. Ninety-one of the 960 households in the six villages were randomly selected using a systematic sampling (every 10^th^) with a goal of enrolling 461 subjects. Sampling frame of each cluster was obtained from the district health office.

### Data collection procedures

After consent and enrollment, blood samples were collected by using Rapid Diagnostic Test (RDT) and blood slides. The laboratory diagnoses were performed following the standard WHO laboratory procedures [24]. Trained laboratory technicians performed finger prick (heel prick for young children) from which they prepared thick and thin blood smears to air dry on the same slide. The smear slides were transported to the nearby health centers for fixation and staining. The thin smear was fixed with methanol solution, and both thin and thick smears were stained with Giemsa solution. The thick smear was used for *Plasmodium* spp. quantification and the thin for *Plasmodium* spp. identification. The negative or positive slides were determined after careful observation of 100 fields of a single slide by experienced laboratory technicians at Tercha Hospital. A laboratory technician at regional laboratory center confirmed each 5 randomly selected low positive and negative blood films according to the current WHO guideline for external quality assurance without prior knowledge to the original results [24].

In addition to blood smear collections, a structured and pre-tested questionnaire was used to collect information from each HH member including associated factors such as background characteristics, mosquito net (MN) use and traditional practices related to malaria infection. Observation of MNs in the HH was also recorded by trained data collectors. Regarding children under five, their parents or guardians were interviewed. Questionnaires were designed in the English language and translated to the local languages (Tsemako and Benegna). All subject questionnaire data and the corresponding smear results were compiled and cross-checked by using subject identification numbers.

### Data analysis procedures

All data were entered into EPI Info version 3.5.3 software for cleaning and then transferred to SPSS version 20 software for analysis. Univariate analysis was used to determine frequencies while an Open Source Epidemiologic Statistics for Public Health (Open Epi) software version 2.3.1 was used to estimate prevalence of malaria and its 95 % CI after cross tabulating by SPSS version 20. Bivariate and multi-variate analyses were performed to examine the association of variables with malaria prevalence. All variables with *p* < 0.2 by bivariate analysis were entered into multivariate analysis. The method used in multivariate model was Backward Stepwise (Likelihood Ratio). The Hosmer and Lemeshow Test was used to assess the fulfillment of basic assumptions of logistic regression while the computed change of -2Log Likelihood and Omnibus tests were used to assess the goodness of model fitness for the variables explained.

### Human subjects and ethics statement

The research was ethically approved by Hawassa University Institutional Review Board and South Omo Zone Health Department. The study participants were clearly explained about risks, discomfort, procedures, benefits, incentives, and issue of confidentiality and safety of the study.

## Results

**Fig. 1:**
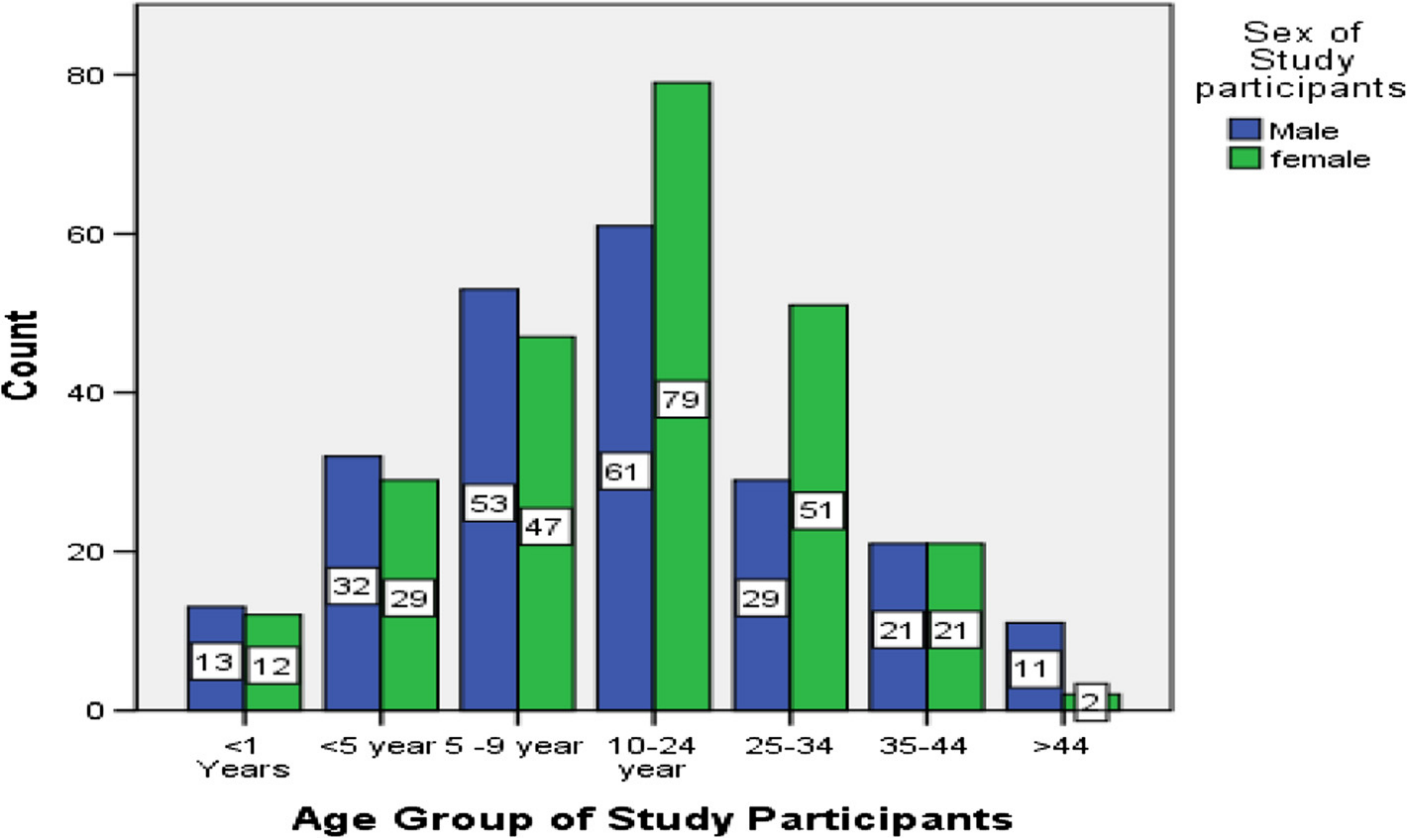
Age group by sex composition of study subjects, BennaTsemay District (a pastoralist Community), South Ethiopia, 2012

A total of 461 participants from 91 HHs provided blood samples and questionnaire data, and no HHs or individuals declined to participate in the survey. The median age of subject participation was 13 years, ranging from 9 months to 65 years. Men were 220 (47.7 %) and women in the reproductive age group were (age 15 to 49) were 129 (28 %), among which 17 (3.7 %) and 18 (3.9 %) were pregnant and lactating women respectively (Fig. 1).

Participants from Tsemay (41.4 %) and Benna (50.1 %) tribes constitute 422 (91.5 %) and 383(83.1 %) were traditional believers (neither Muslim nor Christian by religion). A total of 164 MNs were observed, of which 152 (92.7 %) were LLITNs. The coverage of at least one mosquito net per HH was 90.1 % with the average MN per HH of 1.8. One-hundred-sixty five (39.4 %) of the respondents reported that they were using MN all the time at night and used MN the night before the survey day. However, only 4 (23.5 %) of pregnant and 4 (22.2 %) of lactating women reported that they always sleep under a MN. Two-hundred-nine (45.3 %) of the study participants practiced *‘Rhanto/Rhae*’ while 278 (60.3 %) visited ‘witchcraft’ practitioners (Table 1).

**Table 1:** Background, Mosquito nets utilization and traditional practices of participants among households in BennaTsemay district, Southern Ethiopia, 2012

Characteristics	Frequency	%
Family Education (*n* = 461)		
Primary school attendance	47	10.2
No school attendance	414	89.8
Family marriage type (*n* = 461)		
Polygamous	248	53.8
Not polygamous	213	46.2
Family’s housing type (*n* = 461)		
thatched roof with wall	303	65.7
thatched roof without wall	158	34.3
HHs status (*n* = 461)		
Model HH (trained for Health Extension Package)	271	60.5
Non model HH	190	39.5
Distance of HHs from Mosquito breeding site (*n* = 461)		
Within three kilometres	208	45.1
Greater than three kilometres	253	54.9
Family Technological access (mobile phone, Radio) (*n* = 461)		
Yes	246	53.4
Type of MN (observed) (*n* = 164)		
LLITN (Permanet)	152	92.7
Other (Olyset, Safenite)	12	7.3
Participants’ MN condition (*n* = 461)		
Safe MN (not torn)	152	33
Unsafe MN (torn)	267	57.9
No MN possessed	42	9.1
MNs over sleeping areas (hanged) (*n* = 419)		
Yes	196	46.8
MNs used for other purposes (n-419)		
Yes	169	40.3
Saving MN for later use (*n* = 419)		
Yes	146	34.8
MN owned in use (*n* = 461)		
All the time	279	60.5
Some times	182	39.5
Slept Under MN last night following survey day (419)		
Yes	165	39.4
Boronge’ practice (*n* = 461)		
Yes	291	63.1
Ara’ practice (*n* = 461)		
Yes	293	63.6
Dugo’ house use(*n* = 461)		
Yes	278	60.3
In the past 1 month, performed at least one traditional Practices (*n* = 461)		
Yes	307	66.6

*Footnote*: *MN* mosquito net, *LLITN* long lasting insecticide treated net, *HH* household

### Prevalence of malaria

Among 461 individuals examined for malaria parasites, a total of 28 (6.1 %, 95 % CI = 4.2, 8.4) cases were reported positive for malaria. Out of these, *Plasmodium falciparum*, *Plasmodium vivax* and mixed infection constituted 18 (64.3 %, 95 % CI = 45.5, 80.2), 6 (21.4 %, 95 % CI = 19.8, 54.5) and 4 (14.3 %, 95 % CI = 4.7, 30.9), respectively. Malaria prevalence was 12.0 % (95 % CI = 3.1, 29.3) among infants and 9.8 % (95 % CI = 4.1, 19.3) among children under-five years old. Eight per cent of children aged 5 to 9 years were infected. But, the infection rate among those aged 10 to 24 years (conventionally called youth) was 2.9 % (95 % CI = 0.9, 6.7). A similar rate of malaria infection was observed among the 35 to 44 year old group (2.4 %, 95 % CI = 0.1, 11), while no infection was observed among participants 45 years of age and older. Generally, the rate of infection declined as the age increased; though a significant rise was seen among the 25 – 34 year old age group (7.5 %, 95 % CI = 3.1, 14.9). Stratifying by sex, women and men were infected at comparable rates at 6.6 % and 5.5 %, respectively. However, the infection rate among lactating (22.2 %, 95 % CI =7.5, 45.3) and pregnant (17.6 %, 95 % CI = 4.7, 40.9) women was much higher compared to other segments of the population. The infection rate was higher among those who practiced at least one traditional practice (8.1 %) compared to those who did not (1.9 %) (Tables 2 and 3).

**Table 2:** Malaria prevalence by Ethnic Community and ‘kebeles’, BennaTsemay District, Southern Ethiopia, 2012

Measures	Communities (*n* = 461)		Kebeles (*n* = 461)					
	Tsemay	Benna	*Bori*	*Chali*	*Luka*	*Duma*	*Enchete*	*Alduba*
Frequency	230	231	122	67	69	132	29	42
No. (%)	18(7.8 %)	10(4.3 %)	5(4.1 %)	4(6.0 %)	7(10.1 %)	9(6.8 %)	2(6.9 %)	1(2.4 %)
95 % CI	(4.9, 11.9)	(2.2, 7.6)	(1.5,8.4)	(1.9,13.8)	(4.5,19.0)	(3.4,12)	(1.2, 21.0)	(0.1,11)

**Table 3:** Prevalence of Malaria and potential infection risk factors, BennaTsemay District, Southern Ethiopia, 2012

Factors	Number	Infected cases (%)	95 % CI
Over all prevalence	461	28(6.1)	(4.2, 8.5)
Reproductive age group			
No	332	20(6.0)	(3.8, 9.0)
Yes	129	8(6.2)	(2.9, 11.4)
Religion			
Traditional believers	383	25(6.5)	(4.4, 9.3)
All other religions (Christian and Muslim)	78	3(3.8)	(1.6, 6.1)
Family Education			
Primary school attendance	47	4(8.5)	(2.8, 19.3)
No school attendance	414	24(5.8)	(3.8, 8.4)
HH technological access (mobile, radio)			
Yes	246	5 (2.0)	(0.7, 4.4)
No	215	23 (10.7)	(7.0, 15.4)
Condition of MNs			
Safe MN(good)	152	1(0.7)	(0.03, 3.2)
Unsafe MN(torn)	272	23(8.5)	(5.6, 12.2)
No MN	42	4(9.5)	(3.8, 8.3)
Slept under MNs prior to day of survey			
Yes	165	2(1.2)	(0.2, 3.9)
No	254	22(8.7)	(5.6, 12.6)
‘Boronge’ practice			
No	170	3(1.8)	(0.5, 4.7)
Yes	291	25(8.6)	(5.8, 12.2)
‘Ara’ practice			
No	168	5(3.0)	(1.0, 6.5)
Yes	293	23(7.8)	(5.2, 11.4)
‘Dugo’ house use			
No	183	6(2.3)	(0.7, 4.4)
Yes	278	22(7.9)	(5.2, 11.6)
Witchcraft visit			
No	183	6(2.3)	(0.7, 4.4)
Yes	278	22(7.9)	(5.2, 11.6)
‘Rhae/Rhaento’ practice			
No	252	8(3.2)	(1.5, 5.9)
Yes	209	20(9.6)	(6.1, 11.1)

*Footnote*: *MN* mosquito net, *HH* house hold, *CI* confidence interval

### Infection prevalence associations

The association of selected background variables, mosquito net use and traditional practices related to malaria infection were examined by bivariate and multivariate models. In bivariate models, polygamous marriage (crude Odds Ratio [COR] 5.6, 95 % CI = 1.9, 16.4), living within three kilometers of potential mosquito breeding site (COR 17.9, 95 % CI = 4.2, 76.5), being a lactating woman (COR 5.0, 95 % CI = 1.5, 16.3), irregular or no use of MNs (COR 10.4, 95 % CI = 3.6, 30.6), MNs not over sleeping areas (COR 10.6, 95 % CI = 2.5, 45.8), visiting Witchcraft (COR 2.5, 95 % CI = 1.0, 6.0), practicing ‘Ara’ (COR 2.8, 95 % CI = 1.0, 7.4) and exercising at least one traditional practices (COR 4.5, 95 % CI = 1.3, 15.0) were significantly associated with the malaria infection (*p* < 0.05). However, variables such as religion, ethnic group, age groups, school attendance, pregnancy and sex did not significantly associate with infection of malaria (Table 4).

**Table 4:** Comparison of Malaria infection with risk factors, BennaTsemay District, Southern Ethiopia, 2012

Independent variables	Frequency	Cases		Crude odds ratio (95 % CI)	Adjusted odds ratio (95 % CI)
		No.	%		
Pregnancy status (*n* = 461)					
Other than pregnancy	444	25	5.63	1.00	1.00
Pregnancy	17	3	17.7	3.6 (0 .96, 13.32)	12.6 (1.7, 94.7) *
Saving MN for later use (*n* = 419)					
No	273	2	0.7	1.00	1.00
Yes	146	22	15.4	24.6(5.7,106.4) *	9.6 (2.2, 42.8) *
‘Boronge’ performed					
No	170	3	1.8	1.00	1.00
Yes	291	25	8.6	5.2 (1.6, 17.6) *	
HH status					
Model	189	4	2.1	1.00	1.00
Non-model	272	24	8.8	4.2 (1.4, 12.3) *	
House type					
Thatched roof with wall	158	3	1.9	1.00	1.00
Thatched roof without wall	303	25	8.3	4.6 (1.4, 15.6) *	
Use of MN for other purpose					
No	250	3	1.2	1.00	1.00
Yes	169	21	12.4	11.7 (3.4, 39.8) *	0.4 (0.02, 6.1)
‘Rhae/Rhanto’ performed					
No	252	8	3.2	1.00	1.00
Yes	209	20	9.6	3.2 (1.4, 7.5) *	0 .8(0.2, 2.9)
‘Dugo’ house use					
No	183	6	3.3	1.00	1.00
Yes	278	22	7.9	2.5 (1.0, 6.0) *	1.4(0.1, 16.5)
Modern technology access					
Yes	246	5	2	1.00	1.00
No	215	23	10.7	5.8 (2.2, 15.5) *	1.n0.4, 5.6)

*Statistically significant at *p* < 0.05

Foot note: *CI* confidence interval, *HH* household, *MN* mosquito net

In multivariate analysis, all variables with *p* < 0.2 in bivariate analysis were entered for adjustment and to control for founding. Only pregnancy (adjusted Odds Ratio [AOR] 12.6, 95 % CI = 1.7, 94.7) and saving MN for later use (AOR 9.6, 95 % CI = 2.2, 42.8) were found to be independently associated with malaria infection in the study (Table 4).

## Discussion

This study found that the prevalence of malaria in the pastoralist community was 6.1 % (95 % CI = 4.2, 8.5), and much lower than we had anticipated. However, this rate was similar to recent results reported from different parts of Ethiopia including the National Malaria Baseline survey of 2007 (4.1 %) [12], integrated malaria and trachoma survey of Amhara region (4.6 %) in 2006 [9], and a longitudinal parasitological survey of malaria in high land fringes of Butajira in 2011 (4.4 %) [11]. Furthermore, surveys conducted in 2007 in the SNNPR (5.4 %) and in Jimma town (5.2 %) in 2010 reported comparable findings [5, 7]. However, a study conducted among children under ten years old who live in the villages near Gil Gel Gibe Hydro-Electric Dam II (potential mosquito breeding sites) reported slightly higher prevalence (10.5 %) of malaria [18]. The reason for these consistent findings could be the high coverage of MNs after a massive attention given by the Government of Ethiopia to malaria risk areas like pastoralist communities [14].

Our study has revealed that *Plasmodium falciparum* is responsible for 64.3 % of malaria infections compared to 21.4 % by *Plasmodium vivax* in the study area. This was similar with the result of the National Survey and other studies conducted in Ethiopia [6, 7, 12, 13]. On the other hand, the current finding is not consistent with several reports from a time series study conducted in Jimma town during the years 2000–2009 which reported a higher prevalence of *P. vivax* than P*. falciparum* [5]. One fact that makes these findings different is that the current study was conducted in the Rift Valley areas that are characterized by low lands and high temperatures. In addition, the study area was located near the Kenyan and South Sudanese borders where *P. falciparum* is responsible for 100 % of infections in Kenya and 95 % in South Sudan [1].

In this study, the highest prevalence of malaria was recorded among lactating women (22.2 %) followed by pregnant women (17.6 %) and infants (12 %). Prior studies have not reported prevalence as high as these figures among such vulnerable populations in Ethiopia. One study conducted in Logos, South West Nigeria conducted from March 2007 to February 2008 among pregnant women in an antenatal care program reported a prevalence of 7.7 % (25) which is much lower than the current findings. Among various age groups, under-five years (9.8 %) had the second largest morbidity of malaria next to infants. This result is inconsistent with the findings from a longitudinal parasitological survey conducted in high land fringes of Butajira in central Ethiopia [11]. It is consistent with studies conducted near Gil Gel Gibe Hydro-Electric Power Dam II [18] and a study conducted in 2010 in Jimma town with the same age group (11 %) [5]. However, there were differences in the sampling durations among these studies [5, 11, 18]. The observed high level of malaria infection among women and children might be attributed to low level of immunity among women [12, 13] which could indicate the features of seasonal and unstable malaria. Moreover, another reason could be that this study included a significant number of participants from the households (44.7 %) living within three kilometers of potential sources of mosquito breeding sites, living with thatched roofs without walls (65.7 %), family of polygamous marriage (53.8) and not always using MNs (54.7 %) with relatively low utilization of MNs by HHs of pregnant (23.5 %) and lactating (22.2 %) women in this study area.

Malaria during pregnancy was common among participants of this study, particularly *P. falciparum* which could end up with adverse effects on a mother, fetus and new-born. Affected women may suffer from anemia, fever, cerebral malaria, hypoglycemia and puerperal sepsis and mortality. In regard to mother and fetus health, it commonly causes abortion, still birth and congenital infection while the effect on a new-born leads to low birth weight, pre-maturity, intrauterine growth retardation, illness and mortality [1, 12, 25]. Contribution of malaria towards the morbidity in mother and child health was beyond the scope of the study. Therefore, the vulnerabilities in morbidity and mortality particularly in regards to mother and child health warrants the need of appropriate health care in this community.

The coverage of at least one mosquito net per HH was 90.1 %, of which 92.7 % were LLITNs. The use of MNs prior to the survey day was only 39.4 %, with hanged MNs being observed in 46.8 % of these HHs. This finding is consistent with the result of a study done in Oromiya (91 %) and Amhara regional states in 2007 [16], and higher than the study reported from Buie and Fentalie districts of Ethiopia in 2008 in terms of coverage [19]. However, the use of MNs prior to the survey day was lower compared to the two studies (65, and 68.4 %, respectively) [16, 19]. In addition, those who slept under MNs prior to this survey remained lower despite the higher MNs coverage compared with the results of study conducted by Estifanos Biru Shargie et al. (68.4 and 48.3 %, respectively) [6, 7]. Previous studies indicated that hanged MNs and possession of MNs in the HH did not mean that the participants used MNs [6, 7, 16, 19] which were consistent with this survey’s findings. The high coverage of MNs indicated in this study could be attributed to the increased efforts by the government of Ethiopia in remote areas including pastoralist communities [14]. Despite the government efforts, lower use of MNs in these communities could be due to lack of awareness on the benefits of using MNs [15–17].

Factors such as unavailability of MNs, irregular use of MNs, saving MNs for later use, using MNs for other purpose such as keeping grain, not hanging MNs and unsafe MNs (with multiple holes) were evaluated to determine association with malaria infection. In this study, only participants from HHs who saved (not used MN) MN for later use were 9.6 times more likely to be infected by malaria compared with those who did not save MNs for later use. This result was consistent with the findings reported by Alemu et al. from Jimma town in which those who did not use MNs were 13.6 times more likely to be infected by malaria compared to those who used ITNs [5]. Whereas, the possession of at least one MN in the HH had no association with malaria infection prevalence of the households. This finding supports the argument by Estifanos Biru Shargie et al. that ownership of MNs was not necessarily mirrored with its use [6, 7].

Local traditional practices such as ‘Boronge’, ‘Ara’, ‘Dugo’, witchcraft and ‘Rae/Rhanto’, related to malaria infection were also evaluated. Despite the higher malaria cases observed among performers of traditional practices and a significant statistical association of malaria infection by the bivariate analyses, no significant association was found for such traditional practices by multivariate regression model. This study found that these local practices specific to the study area were not favoring malaria infection. However, attention must be given while performing these practices as higher infection rate was observed among performers of at least one traditional practice (8.1 %) compared to non performers (1.9 %) in the study area.

Since a cross-sectional study design was used in this study, the results cannot be generalized. Information bias was limited by providing orientation for study participants through local languages, training interviewers and blinding blood sample examiners. With these, accuracy was assured and information bias was reduced.

## Conclusion

In spite of good coverage of MNs, malaria in pastoralists communities predominantly affects lactating and pregnant women, infants and under five children. Information, Education and Communication approaches should strongly and regularly be undertaken in a way that it could favor MNs utilization in the pastoralist communities with priority to high risk groups through close monitoring and evaluation at each level of health administration.

## Abbreviations

ACT: Arthimesine combined therapy
DHS: Demographic & health survey
HH: House holds
HU: Hawassa University
ITNs: Insecticide treated nets
IRS: Indoor residual spray
LLITNs: Long lasting insecticide treated nets
MDG: Millennium development goal
MIS: Malaria indicator survey
MN: Mosquito Net
RDT: Rapid diagnostic test
SNNPR: Southern Nations Nationalities People’s Region
